# Four Days Exposure And Reprocessing Therapy For PTSD

**DOI:** 10.1192/j.eurpsy.2023.1019

**Published:** 2023-07-19

**Authors:** O. Duek, I. Levy, I. Harpaz-Rotem

**Affiliations:** 1 Psychiatry, Yale University School of Medicine, New-Haven, United States; 2 Epidemiology, Biostatistics and Community Health Sciences, Ben Gurion University of the Negev, Beer Sheva, Israel; 3 Comparative Medicine, Yale University School of Medicine, New-Haven, United States

## Abstract

**Introduction:**

Post-traumatic stress disorder (PTSD) is a debilitating disorder affecting approximately 6% of the population. Current treatments have been shown to efficaciously reduce symptom burden between 30%-50%. However, due to the high intensity of treatment over a long period of time. drop-out rates are as high as 50%.

**Objectives:**

Assess the effect of one-time ketamine infusion in subanasthetic dosage on PTSD psychotherapy

Assess feasibility and effect of massed, four days, esposure focused psychotherapy for PTSd

**Methods:**

Here, we tested the efficacy of a four-day exposure and processing-focused psychotherapy at reducing PTSD severity. Twenty-seven participants with chronic PTSD were randomized to two groups, one receiving a one-time infusion of ketamine in a subanesthetic dose (0.5mg/kg for 40 minutes), the other receiving midazolam. Both groups underwent four 90-120 minutes of daily psychotherapy sessions a day after infusion, followed by in-vivo exposure practice. The severity of PTSD was assessed with the PCL-5 before and at the end of treatment, and at 30 and 90 days follow-up. Brain reactivation to the trauma reminders was measured using fMRI

**Results:**

PTSD severity in both treatment groups decreased by 13, 16, and 15 points on the PCL-5 at the end of treatment, 30 days follow-up, and 90 days respectively, surpassing the minimum clinical difference of 7.9 points. There was no significant difference in symptom reductions between the treatment groups. However, brain reactivation to trauma stories differed between the groups, with the ketamine group showing a decline in the amygdala and hippocampus reactivation compared to the midazolam group, at the end of treatment.

**Image:**

**Figure:**
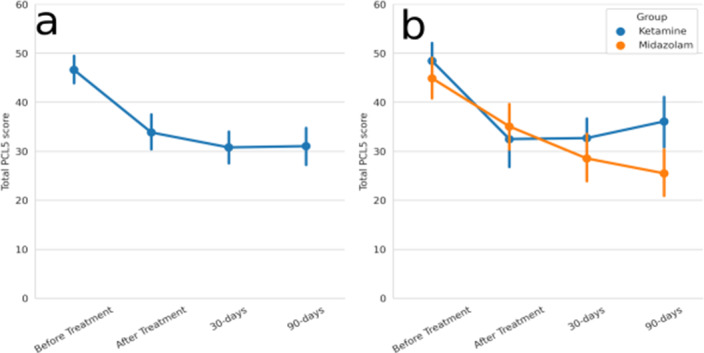

**Conclusions:**

Our results imply comparable efficacy of this short-term intervention to standard trauma-focused psychotherapies, emphasizing its clinical usefulness as a short and effective intervention.

**Disclosure of Interest:**

None Declared

